# RISKS AND VULNERABILITIES AFFECTING ADVANCED CARE PLANNING FOR OLDER ADULTS INVOLVED IN ADULT PROTECTIVE SERVICES

**DOI:** 10.1093/geroni/igz038.646

**Published:** 2019-11-08

**Authors:** Joy S Ernst

**Affiliations:** Wayne State University School of Social Work, Detroit, Michigan, United States

## Abstract

Adult protective services (APS) workers regularly engage in advanced care planning (ACP). This qualitative content analysis of 21 APS cases sought to extend knowledge and uncover characteristics that influenced the ACP process and increased vulnerability of older adults in cases of neglect. The case narratives were examined to identify themes of vulnerability and risk related to ACP. Vulnerabilities of the older adults included multimorbidities, geriatric syndromes, and functional limitations. Risks involving caregivers included health problems and cognitive impairment. Caregivers were overwhelmed or lacked insight into the older adults’ needs. Involvement of other family members was detrimental or non-existent while some advocated for improvements where the caregiver refused assistance. Findings support the development of methods of ACP, including the facilitation of difficult conversations, that would respect the autonomy and dignity of the older adult while meeting the multiple needs of caregivers and family members.

